# Supplementary material for: Are carbon emissions trading and green financial instruments synergistic? -Comprehensive quantitative research based on content analysis
Source: PLoS One. 2024 Mar 7;19(3):e0298601. doi: 10.1371/journal.pone.0298601 (PMC10919601; doi:10.1371/journal.pone.0298601)
Supplement: S1 Data — (ZIP) [file pone.0298601.s001.zip › Data/地级市Z_F工作报告.zip!/╝¬┴╓/╦─╞╜╩╨/└┤╫╘╣1⁄2╓┌║┼í╛┬φ┐╦╩2╛▌═°í┐_2003.docx]

各位代表：

　　我代表市人民政府，向大会报告工作，请予审议。

　　一、2002年政府工作回顾

　　即将过去的一年，在市委的正确领导下，市政府认真贯彻落实“三个代表”重要思想，继续围绕“工业立市、农业增效、财政翻身”三项主要任务，与全市人民一道，与时俱进，开拓创新，艰苦奋斗，真抓实干，经济和各项社会事业取得了显著成绩。

　　国民经济发展势头强劲，工业、农业、财政同步快速增长。预计全市国内生产总值完成201亿元，同比增长13%以上。一、二、三产增加值同比分别增长10%、15%和14%。工业经济运行质量和效益显著提升。预计规模以上工业增加值完成32亿元，同比增长15%。地方工业企业盈利超过8000万元，实现了历史性突破。十大工业基地建设初具规模，重点企业进一步做大做强。企业技术改造、改组、改制力度进一步加大，启动双停企业取得新的明显成果，一批老企业焕发生机。农村经济全面健康发展。粮食生产保持稳定，总量1443万吨。种植业结构调整、水利基础设施建设实现历史性跨越。财贸经济持续发展。预计全市一般预算全口径财政收入可以完成13亿元以上，同比增长8%以上，其中市本级财政收入可以完成6亿元以上，同比增长13%以上。预计全市各类金融机构存款、贷款余领比年初分别增长13.9%和6.6%。预计社会消费品零售总额达到70.8亿元，同比增长12.2%。

　　项目建设取得重大突破，一批重点工程开工、建成、投产。预计全社会固定资产投资完成40.5亿元，同比增长25%。项目年活动取得明显成果，新项目、大项目开发势头强劲。全市新开工建设和续建的重点工业项目达104个，其中投资超亿元的23个，重点培育10个利税超亿元的大项目。今年已有46个较大项目建成投产。红嘴集团30万吨大豆和10万吨花生加工、通钢集团3万吨钢绞线、巨能公司1500万瓶大输液、方向机马自达桥车转向节总成、庆达DVD新生产线等一批重点项目的快速建成和投产，形成了工业经济新的增长点。公路建设实现历史性突破，全年共完成投资5.8亿元，超过了“九五”期间的投资总和。年初安排的46个重点建设项目完成投资20亿元，四白一级路、西辽河大桥、梨树污水处理厂等12个项目相继竣工，东西辽河治理、二龙山水库除险加固等34个项目进展顺利。

　　农村经济结构调整迈出实质性步伐，现代农业发展模式初步形成。以强市富民为目标，以工业拉动为重点，以长平经济走廊和长郑经济带为主要载体，大力推进农村工业化、市场化、集约化、城镇化和农业基础设施现代化。农产品加工龙头企业带动能力显著增强。专用粮食生产和订单农业比重不断扩大，棚膜经济、牧业小区、商品经济林蓬勃发展，农业产业化水平明显提高。农村劳务经济发展取得重大突破，全年共组织劳务输出35万人。小城镇基础设施日趋完善，城镇化水平进一步提高。长平经济走廊和长郑经济带的农业生产经营方式、农村经济结构、农民生活方式和农村工作的领导方式发生深刻变革，农业资源使用效率和农业生产组织化、社会化程度显著提高，市场农业、现代农业、效益农业显现出良好前景和巨大潜力。

　　开发区建设实现超常规发展，各项改革取得新突破。六个开发区新开工项目115个，到位资金10.16亿元。其中投资千万元以上大项目41个，到位资金7.25亿元。基础设施投入1.54亿元，道路和水、电、气等配套设施明显改善。四平经济开发区、红嘴高新技术开发区平均每月上一个新项目。红嘴高新技术开发区和范家屯开发区被批准为省级开发区。全市招商引资项目612个，到位资金33.63亿元。浙江纳爱斯、福建耀华、通钢集团、吉林油田等一批大型企业集团的进入和扩大投资，为四平经济注入了强大活力。国土资源市场化配置取得重要进展，建立了国有土地收购储备库，成功地进行了国有土地使用权招标拍卖。住房制度改革稳步推进，公积金管理体制改革进展平稳。城镇职工基本医疗保险和医药卫生体制“三项改革”继续深化。农村税费改革试点工作积极推进，全市平均减负20％左右。对外联系、交流与合作不断加强。出口创汇增势强劲，预计全市出口额完成1932万美元，同比增长25%。

　　税收质量进一步提高，财政形势明显好转。按照发展税源型、就业型、可持续型经济的要求，稳定基础税源，开发潜在税源，培育新兴税源，壮大骨干税源。实行国税保基数、地税保财力、财政保平衡的对策，今年财政状况进一步好转，财政供养人员工资基本正常发放，“两个确保”和城市居民最低生活保障的财政支撑能力增强，重点事业发展得到保证。同时通过涵养税源、培植税源，后续税源明显增多，骨干税源正在形成，财政后劲明显增强。

　　基础设施与环境质量明显改善，城市面貌发生巨大变化。全年在建房屋施工面积105万平方米，建设了海银绿苑、阳光新城等一批高标准智能化住宅小区。中心城市规模不断扩大。新建扩建英雄桥、南迎宾桥、北迎宾桥等6座桥梁。改造打通南北迎宾街、青年街等15条主次干路，完成了中央东路延伸工程，城市道路网络体系进一步完善。城市供水、供热、供电、燃气等公用服务功能不断加强。开工建设了北沟、宏宇等8个市场。英雄广场一期工程竣工，正式向市民开放。南河改造工程完成561延长米，累计完成2200延长米。站前广场改造工程正式开工建设。城区植树16万株，庭院绿化三年达标任务基本完成，建成区绿化覆盖率达到35.24%，人均公共绿地面积6平方米，绿化、美化、亮化水平显著提高。城市管理不断强化，省级卫生城市的成果得到巩固和发展。

　　社区建设成绩斐然，被评为全国社区建设示范市。市区新建、改建、扩建社区综合用房79处，建筑面积3.36万平方米，社区普遍设立了各类组织机构，选聘了工作人员，配备了微机、电视、电话等现代办公设备，75%以上的社区配备了体育健身器材和室外文体活动场所，在全国率先实现了社区建设覆盖全城的目标，初步形成了城市基层管理、居民服务和民政工作三个平台。社区的文化、体育等活动蓬勃开展，便民服务、救助保障、医疗保健、社区安全、居民教育等社区功能逐步完善。全国社区建设现场会在平成功召开，我市被评为全国社区建设示范城市。

　　民营经济快速健康发展，成为国民经济的重要组成部分。预计全市民营经济实现增加值96亿元，同比增长71.4%，占全市国内生产总值的比重达到47.8%。纳税100万元以上的民营企业达到30户。在规模以上工业企业中，民营工业达到146户，占46.5%；实现增加值15.7亿元，占47.6%；上缴税金2.7亿元，占30.5%。民营经济投资预计占全市固定资产投资的50％以上，在经济发展中发挥着越来越重要的作用。

　　各项社会事业全面进步，精神文明建设取得可喜成绩。全年引进推广重点科技成果16项，科技进步对经济增长的贡献率达到47%。完善了基础教育管理体制，师德和行风建设进一步加强。中小学生流失现象得到有效控制，实现了公办教师工资县级统筹发放。卫生医疗监督服务质量得到提高，农村卫生服务明显加强。文化活动丰富多彩。广播电视基础设施得到根本改善，保证了安全优质播出。成功地举办了首届社区（城区）体育大赛，群众体育位居全国先进行列；竞技体育取得了省第十四届运动会夏季项目第二名的好成绩。较好地实现了控制人口增长和提商人口素质的目标，人口自然增长率控制在7.68‰以内。群众性精神文明创建活动成效显著，被评为全国创建文明城市工作先进城市。旅游基础设施不断完善，环境保护和生态建设富有成效，双辽生态示范区建设取得阶段性成果，可持续发展能力明显增强。人民防空工作取得新进展，防震减灾水平进一步提商。残疾人、老龄、史志、档案、双拥等工作也都取得了新的进步。

　　人民生活条件继续改善，生活水平不断提高。预计城镇居民人均可支配收入超过5200元，农民人均纯收入达到2530元，同比分别增长23%和13.9%。社会保障体系逐步完善，养老、失业保险覆盖面均达到100%。新开发就业岗位3万个，安置下岗失业人员2.15万人。“心连心”帮贫扶困活动不断深入，机关三项重点工作全面开展，解决了部分群众的住房、再就业等生活困难问题。城镇和农村居民人均住房面积分别达到13.8平方米和19.3平方米。固定电话持续增长，移动电话正在迅速普及，邮政业务总量同比增长12%。城乡市城繁荣，商品供应充裕，人民群众的衣食住用行都有较大改善。

　　民主法制建设得到加强，社会保持全面稳定。各级政府自觉接受人大的法律监督、工作监督和政协的民主监督，办复人大代表建议50件、政协委员提案165件。强化行政复议、行政执法监督检查，制定规范性文件11件。政府机构改革顺利完成，领导干部廉洁自律、查处违法违纪案件、纠正部门和行业不正之风等工作进一步深化，整治经济发展软环境工作不断深入，提高了机关办事效率和服务水平。统计、审计、物价、技术监督和工商管理工作继续加强，进一步整顿和规范了市场经济秩序。民族、宗教和侨务工作取得新进展。深入开展“四五”普法教育，全民法律意识和法制观念逐步增强。严打整治斗争走在全省前列，同法轮功邪教组织斗争卓有成效，社会治安长效机制建设成果显著。信访工作全面加强，妥善解决了一批事关群众切身利益的实际问题，实现了政治安定和社会稳 在肯定成绩的同时，我们也清醒地认识到，我市经济和会生活中还存在一些矛盾和问题。主要是：经济总量不足，结构不优，效益不高，外向度不够；工业企业的整体素质和竞争力有待提高；农业基础设施仍然薄弱，农业增效任务繁重；一些县市财政困难仍然很大，财政收入的增长满足不了刚性支出的需要；经济发展环境尚需进一步改善；下岗职工和受灾地区群众及部分农民的生活仍然比较困难；社会保障体系不健全，再就业压力很大，社会不稳定因素仍然存在；政府工作与经济发展还有不相适应的地方，与上级的要求和群众的愿望还有差距，官僚主义、形式主义和某些腐败现象在一定范围内还不同程度地存在。这些问题已经引起我们的高度重视，需要付出艰苦的努力，采取有针对性的措施加以解决。

　　各位代表，即将过去的一年，我市经济形势发生重大变化，工业立市取得显著成效，建设经济强市迈出扎实步伐，两个文明建设协调发展，全市政治安定，社会稳定，政通人和，百业俱兴。这是我们解决贯彻落实中央和省委、省政府的路线、方针、政策的结果，是市委明确发展思路、抓住主要矛盾、科学决策、正确领导的结果几是市人大、市政协等吝方面大力支持和帮助的结果，是各地、各部门开拓进取、求真务实和全市人民同心同德、艰苦奋斗的结果。在此，我代表市人民政府，向全市广大工人、农民、知识分子、各民主党派、工商联、人民团体和离退休老同志表示崇高的敬意！向关心和支持四平建设的中省直单位、驻平部队、武警官兵和国际友人，表示衷心地感谢！

　　二、2003年主要工作任务

　　各位代表，党的十六大明确提出，要紧紧抓住21世纪头20年这个大有作为的重要战略机遇期，全面建设惠及十几亿人口的更高水平的小康社会，使经济更加发展、民主更加健全、科技更加进步、文化更加繁荣、社会更加和谐、人民生活更加殷实。对四平而言，更具有现实紧迫性和历史重要性。我们务必紧紧抓住机遇，把四平的各种优势最大限度地发掘出来，加快发展步伐，构筑主导产业，扩充经济规模，提高经济质量，使四平在全面建设小康社会的进程中全面创业、快速崛起。

　　明年政府工作的总体要求是：全面贯彻党的十六大和市委四届二次全会精神，高举邓小平理论伟大旗帜，以“三个代表”重要思想为指导，按照全面建设小康社会和建设工业省、科技省、生态省的要求，坚持“工业立市、科教兴市、开放带动、县域突破、人才兴业”战略和“高效益、广就业、可持续”方针；继续以工业立市为重点，突出工业立市、农业增效、财政翻身三项主要任务；继续实施扩大中心城市规模、建设长平经济走廊、培育十大工业基地、扶持壮大企业集团、搞好六个开发区建设的战略布局；发展税源型、就业型、可持续型经济，加快工业化、信息化、城镇化和经济国际化进程，加强精神文明、政治文明建设，促进经济快速发展和社会全面进步，争取早日实现建设经济强市目标。

　　宏观调控的主要预期目标是：国内生产总值增长12％左右，其中第一产业增加值增长8%,第二产业增加值增长14.5%,第三产业增加值增长15%；全社会固定资产投资增长16％左右；财政收入增长8％以上；社会消费品零售总额增长9%;城镇居民人均可支配收入增长10％左右，农民人均纯收入增长9％左右；城镇登记失业率控制在4.5％以下；人口自然增长率控制在8.3‰以下。

　　为完成上述任务和目标，要重点抓好以下工作：

　　（一）以全力推进工业立市为重点，继续在项目开发、企业改制、资本运营、搞好重点难点企业、发展高新技术和民营工业六个方面寻求突破，走新型工业化道路。

　　继续把项目开发摆在突出位置。按照大规模、高科技、外向型、新机制、可持续发展的要求，全面落实230个重点工业项目，切实抓好84个十大工业基地项目，继续重点培育10个利税超亿元的重大工业项目。积极发展高新技术产业和新兴产业，用信息化带动工业化，用高新技术、先进适用技术改造传统产业和骨干企业，加快技术应用和产品开发，不断提高产业层次和技术装备水平，走科技含量高、经济效益好、资源消耗低、环境污染少、人力资源优势得到充分发挥的新型工业化路子。重点抓好巨能4000万瓶大输液、黄龙20万吨淀粉糖、耀华700吨级优质浮法玻璃等20个较大的技改项目，确保实现技改投资10亿元以上。抓紧做好双辽电厂二期和四平热电厂二期工程准备、接续工作。加强项目管理，强化组织调度，确保重点项目建设的进度和质量。

　　切实加大企业改制工作力度。积极探索和完善国有资产经营体制和方式，实现国有资产保值、增值。继续推进以产权制度改革为核心的国有企业改革，推动企业体制、机制、技术和管理的创新。鼓励和吸引各类组织、法人及域外投资者等投资入股．大力发展混合所有制经济。推动企业内部股权转让，支持经营者持大股，解决企业机制不到位的问题。积极扶持股份公司上市融资，重点抓好四平宏宝莱股份有限公司等企业的上市工作。继续推进三项制度改革，建立经营者能上能下、职工能进能出、收入能增能减的新机制。全力启动“双停”企业，努力搞活经营，逐步摆脱困境。对资不抵债、扭亏无望的亏损大户和空壳企业，在安置好职工的前提下，依法实施破产。

　　进一步壮大优势企业。坚持以市场为导向，按照规模经济的发展要求，切实把红嘴集团、烟厂、纳爱斯、耀华、巨能、方向机、庆达、埃默等一批企业做大做强。在粮豆深加工、肉食加工、能源、饮品、换热器、金属材料及制品、行走机械及汽车零部件、高新技术产业、医药化工、玻璃等领域形成一批具有比较优势和发展前景的企业群体，进一步加快十大工业基地建设。抓住国家颁布实施中小企业法的契机，鼓励和支持我市众多的中小企业加快发展，着力放开搞活。加强中小企业与大公司、大集团的配套与合作，提高行业、产业及企业间的联系度，推动中小企业向“专、精、特、新”方向发展，着力培育一批“小巨人”企业。

　　（二）以长平经济走廊和长郑经济带为示范，促进农村经济的工业化、市场化、集约化、城镇化和农业基础设施现代化，努力实现农业增效。

　　坚持用工业思维谋划农业发展，推进农业产业化进程。强化基地建设和订单生产，重点在粮豆、畜产品、瓜果菜精深加工上做文章，建设一批立市、立县、立乡的龙头企业，拉长农业产业链，提高产业化发展水平。加强农畜产品专业市场和批发市场建设，鼓励发展各类中介服务组织，扩大经纪人队伍，健全农村信息网络，搞活农产品流通。

　　狠抓牧业小区和棚膜经济，发展规模畜牧业和优质特色农业。新增牧业小区100个，每个乡镇及有条件的村都要建1至2个枚业小区，形成集约生产优势。调整畜群品种结构，搞好防疫和卫生安全检疫，提高畜产品市场竞争力。继续增加专用玉米、大豆等优质农产品种植比重，集中发展棚膜经济，年内新增大棚、温室面积5000亩，地膜2万亩。

　　加强基础设施建设，改善农业生产条件。继续搞好水库除险加固，提高防洪除涝能力。新打农田井5000眼，新增灌溉能力50万亩、坐水种能力80万亩。大力发展草业经济、林地经济，结合“一亩效益田”工程发展井旁经济。继续做好人工增雨、防雹等防灾减灾工作。制定和完善农产品生产标准和技术规程，建立农产品质量监测检验体系和质量认证体系，推进农业标准化进程。加快发展无公害、绿色、有机食品等优质名牌农产品，绿色食品基地面积达到60万亩。大力普及种植、养殖、加工、包装、储藏等先进适用技术，提高科技在农业增效中的贡献率。

　　继续推进小城镇建设，加快城镇化进程。调整小城镇发展战略，优先发展长平经济走廊和长郑经济带沿线城镇，把有辐射能力的中心城镇、历史老镇和周边重镇做大做强。创新小城镇管理体制，积极推进财政体制、投融资体制、土地流转制度和户籍制度改革。加紧实施各级试点镇联网工程，对小城镇实行动态管理。

　　深化农村改革，保持农村稳定发展的良好局面。搞好农村税费改革收尾工作，完善各项配套改革，切实减轻农民负担。全面推行“村帐乡管”，提高农村财务管理水平。积极推进农村经济组织创新，鼓励以专业合作社、专业协会、公司加农户、股份合作经营等形式，推动农村经济实现新的发展。

　　（三）以开发区建设为载体，继续加大招商引资工作力度，开创开发开放工作新局面。

　　大力抓好开发区建设。继续多方筹措资金，加强基础设施建设，提高吸引力和承载力。以发展高新技术产业为重点，突出抓好项目开发，争取更多的投资，实现快速发展。坚持封闭式管理、开放式运行，使工作机制与国际惯例接轨，提高管理和服务水平。

　　努力扩大招商引资领域和规模。坚持“引进来”和“走出去’，相结合，突出项目招商，落实招商引资责任制，开创招商引资新渠道。积极参加各类招商引资活动和商品展洽会，开发推介招商项目。坚持抓住重点项目、组成专门班子、落实贵任分工、工作一抓到底的措施，在重点项目招商上实现新突破。

　　注重发挥外贸出口对经济的拉动作用。继续开发和培育国际市场，鼓励和支持各类企业扩大出口，提高高新技术产品和机电产品出口比重。进一步加强对外经济合作，鼓励有条件的企业到境外投资办厂，扩大劳务输出和加工贸易，不断拓宽发展空间。

　　（四）以民营经济为新的增长点，全力营造成长发育空间，倾心扶持壮大民营企业。

　　营造宽松的社会环境。把发展民营经济放在工业立市和建设经济强市的突出位里．真心支持，倾力扶持。落实对民营企业的“国民待遇”，在信贷、技改资金、税收、项目审批、土地使用、劳动用工、人才管理、社会保障等方面，与其它企业一视同仁，为民营经济创造良好的外部条件，提供广阔的发展空间。树立创业典型，搞好创业培训，发展壮大民营企业家队伍。 保护民营企业合法权益。对在四平市境内创业和发展的民营企业，做到政治上给地位，社会上给名誉，权益上给保护。行政执法部门对民营企业的检查内容、执法程序、立案办案依据都要进行公示和规范，做到公开、公平、透明。对规模企业、高新技术企业和纳税大户实行封闭管理，重点保护，严禁乱检查、乱收费、乱罚款，使民营企业能够放心、放胆发展。

　　形成民营经济竞相发展态势。一是鼓励民营企业通过收购、兼并、租赁等形式，参与国有企业和集体企业资产重组，转制一批；二是对基础好、有潜力的民营企业，重点保护，全力支持，发展壮大一批；三是在发展县域经济中，引导广大农民发展二、三产业，开发创办新上一批；四是在城镇下岗职工中，积极落实再就业政策，支持鼓励自办一批；五是通过招商引资，利用优惠政策、优良环境、优质服务，吸纳引进落户一批；六是鼓励有胆有识的干部走出机关，下海领办创办一批；七是通过发展专业市场和批发市场，搞活商贸流通，实行前店后厂、带动培育一批，使民营企业在经济发展中发挥更加重要的作用。

　　（五）以实现财政税收的良性循环为主要任务，大力发展税源型经济，努力搞好增收节支。

　　进一步加大管理和改革力度。积极推行部门预算，全面清理银行帐户，实行预算内外资金统管。加大对国库对帐检查、罚没收入、行政事业性收费、政府性基金等监管、稽查和审计力度。加强对各种债务资金的监督管理，增强项目的自身偿债能力。对重点财政支出项目和单位进行专项检查。

　　大力增加财税收入。继续贯彻“加强征管，堵塞漏洞，清缴欠税，惩治腐败”的方针，重点抓好主体税种和税源大户的征管，深入开展对集贸市场、加油站税收的专项整治，积极清理欠税，做到应收尽收。稳定基础税源，开发潜在税源，培植新兴税源，壮大骨干税源，为增强财政支撑力奠定坚实基础。

　　严格控制财政支出。按照“一要吃饭，二要建设”的方针，重点落实“两个确保”和城市“低保”资金，保证教师和机关事业单位工资按时发放。加强预算管理，严格执行支出预算，除救灾等少数急需的特殊支出外，一律不再追加新的支出项目。

　　金融部门要加大对经济发展的支持力度。合理运用货币政策，积极组织存款，增加信贷资金可供量。认真落实支持中小企业、再就业、扩大需求及启动消费的信贷政策，积极开展中小企业信贷担保和农户小额信用贷款，改进和完善金融服务。进一步强化金融监管，采取积极有效措施，防范和化解金融风险。

　　（六）以扩大中心城市规模为目标，进一步加强城乡建设和管理，积极创建国家级卫生城市。

　　调整完善城市总体规划。按照“突出功能、合理布局、优化结构、组团发展”的要求，充分考虑扩大中心城市规模的需要，市区主要向东、向北发展，适当向西发展，控制向南发展。四平经济开发区和红嘴高新技术开发区规划面积都要达到10平方公里以上。进一步完善近期建设规划和市域城镇体系规划，提高城市品位，塑造城市形象，展现四平的城市文化特色。

　　继续加快城乡基础设施建设。重点抓好以下几项工作：一是继续加大旧城改造力度，加快站前广场建设，完成站前人防工程。二是继续完善交通路网。以建设国省主干线和市区环城路为重点，完成交通基础设施投资8.4亿元，实现乡乡通油路目标。市政道路建设重点完成北迎宾街公铁立交桥、南湖路西段、英雄大街西段延伸工程以及一批城市主次干道的维修改造，核心区道路硬覆盖率达到100%。三是搞好城市广场及部分街路灯光景观工程建设。全面完成英雄广场二期工程。继续推进南河河堤改造工程。四是完善有形市场体系建设。改建扩建粮油、农业生产资料等6个专业批发市场和爱民、东郊等7个较大规模的农贸市场，积极筹建和规范室内劳动力市场。五是加快环境保护基础设施建设。完成污水处理厂一期工程，开工建设南北河污水截流干管工程。积极创造条件，尽快建设垃圾无害化综合处理厂。六是加快园林绿化建设。完善城市园林绿化体系，启动环城绿化带工程，力争城市建成区绿地率达到37%以上，人均公共绿地面积7平方米以上。

　　全面提高城市管理水平。继续深化城市管理体制改革，努力提高综合执法管理效能。整顿交通秩序，提高畅通工程建设水平。推行全天候保洁制度，取缔露天垃圾点，垃圾清运做到日产日清。拆除违法违章建设，规范建设工地管理，落实“门前五包”责任制，整治占道经营，规范广告牌匾，完成城市标准化地名标志设置。强化污染物排放总量控制，加大对工业污染源及各类服务行业的监管力度，逐步提高城市总体环境质量。加大住房公积金清欠、征缴和管理力度，深化住房制度改革，继续改善中低收入家庭的住房条件。

　　（七）以加强基层基础建设为目的，不断完善社区功能，积极开展社区活动。

　　切实加强社区管理。理顺社区内外关系，转变社区居委会工作职能，提高社区干部政治素质和工作水平。增加各级财政投入，保证社区专职人员工资补贴。制定和完善各类管理细则和实施方案，为社区工作提供有力的制度保障。深入开展创建文明社区活动．组织社区居民开展评议居委会成员和评医、评警活动，扩大基层民主，增加工作透明度，提高社区居民参与意识，增强认同感和归属感。

　　不断提高社区服务标准和水平。积极开展疾病预防、医疗保健、计划生育指导等社区卫生服务，建立社区居民健康档案。教育、文化、体育等部门要定期举办社区赛会，组织引导群众开展丰富多彩、健康有益的社区活动。全面开展杜区警务工作，提高综合执法和服务水平。开展经常性、群众性的法制宣传教育，为居民提供法律援助、法律咨询服务。组织动员更多的老同志参与关心下一代工作。大力开展绿化、美化，改善社区环境。推进社会救助、社会福利和社会保障服务，为特殊群体提供更好的生活条件。

　　大力发展社区经济。广泛动员社会力量，通过共驻共建、资源共享，解决好杜区设施维护和日常办公经费来源。鼓励社区创办以安置下岗职工再就业为目的的经济实体，强化“费随事转”原则，为社区建设提供必要的补助。落实鼓励社区经济发展的各项优惠政策，完善社区服务网点，培植社区经济支撑点。

　　（八）以扩大就业和再就业为根本途径，提高社会保障能力，切实改善人民生活。

　　努力做好再就业工作。一是通过加快经济发展，拉动再就业。大力发展第三产业，拓展服务就业空间；积极发展个体私营经济，扩大非公有制经济的就业容量；全力扶持中小企业和劳动密集型企业发展，创造就业比较优势。全年新开发就业岗位4万个。二是通过组织劳务输出，扩大再就业。在搞好农村劳务输出工作的同时，积极探索城镇下岗失业职工劳务输出的新办法、新途径、新措施，实现下岗失业人员就业的新突破。三是通过加大政策支持力度，带动再就业。认真落实各项就业和再就业政策，对就业困难的下岗失业人员实行再就业援助，增加财政预算支出，提供再就业保障资金。四是通过完善服务体系，促进再就业。加强劳动力市场信息化建设，及时为下岗失业人员提供免费职业介绍；建立健全基层就业服务组织，把就业和社会保障服务体系向街道、社区延伸，通过社区服务、社区管理拓宽就业岗位，通过发展社区经济拓宽就业岗位；大力开展职业技能培训，免费为下岗人员进行再就业培训，增强再就业本领。加强劳动监察仲裁，整顿规范中介机构，为下岗失业人员创造公平、安全的就业环境。

　　深入细致地做好“两个确保”工作。继续坚持“三三制”原则，多渠道筹措资金，确保进中心下岗职工、暂时还难以解除劳动关系的下岗职工及时足额领取基本生活费。进一步加大养老保险基金的征缴、扩面和清欠工作力度，着力解决好破产企业职工、解除劳动关系人员和灵活就业人员的参保、续保问题。继续坚持社会统筹与个人帐户相结合的企业职工基本养老保险制度，企业退休人员逐步纳入社区管理，积极推进社会保险管理服务的社会化。

　　切实搞好解困救济。完善城市居民最低生活保障制度，建立“低保”与临时救济、政策扶困、社会扶助相结合的城市扶困机制，确保符合条件的低保对象及时得到各方面的救助。加强救灾救济工作，解决好冬令和春夏荒期问灾民的口粮。

　　（九）以社会主义精神文明建设为动力，全面实施科教兴市战略，积极发展各项社会事业。

　　以进入全国文明城市行列为契机，广泛深入开展群众性精神文明创建活动。认真贯彻公民道德建设实施纲要，弘扬爱国主义精神，以为人民服务为核心、集体主义为原则、诚实守信为重点，加强社会公德、职业道德和家庭美德教育。在全市形成爱国守法、明礼诚信、团结友善、勤俭自强、敬业奉献的良好社会风尚，提升城市整体文明程度。

　　坚持教育优先发展的战略地位，促进各级各类教育协调发展，不断提高办学水平和教学质量。突出抓好职业教育管理体制改革，加强政府统筹和社会参与，促进职业教育与经济社会发展的紧密结合，实现我市职业教育的新突破。大力实施科教兴市战略、可持续发展战略和人才兴业战略，积极培育科技创新人才，建设全方位、高素质、社会化的人才队伍。稳定低生育水平，提高人口素质。继续推进城镇职工基本医疗保险和医药卫生体制改革。大力加强农村卫生工作，努力提高农民的防病治病水平。切实搞好公共场所卫生和食品卫生管理工作，加大城乡“除四害”工作力度，防止重大疫情的发展。推进文化产业发展和文化市场管理，加强和改进广播电视工作，不断满足群众文化生活需求，为经济建设和社会发展营造良好的舆论环境。积极贯彻群众体育和竞技体育协调发展的基本方针，大力发展体育事业。

　　（十）以社会稳定为前提，加强社会治安综合治理，努力化解和妥善处理各种社会矛盾。

　　进一步加强社会治安综合治理。继续严厉打击各类黑恶势力，大力开展破案攻坚和追逃工作，适时开展专项斗争，坚决压住治安阵脚。严厉打击各类锋芒性犯罪，把群众反映强烈的严重威胁社会治安的突出问题解决好。建立和完善公安机关的快速反应机制，健全社会治安群防群治体系，不断探索警务改革，充分发挥社区警务在震慑犯罪、预防犯罪和服务群众方面的积极作用。密切注视、严格控制邪教组织的动向，有效防范和惩治邪教组织的破坏活动。

　　努力化解和妥善处理各种社会矛盾。针对不同种类的问题和矛盾，采取不同的方法区别对待，认真加以解决。对应该解决并可以解决的，要立即研究解决；对应该解决并经过努力能够解决的，要积极创造条件，下决心解决；对应该解决但一时难以解决的，也要及时深入到群众中去，做深入细致的思想政治工作，求得群众的理解和支持；对不合理或无理要求的，要坚持原则，讲清道理。对待群体性事件，总的原则是以说服教育和疏导为主，防止演变升级。对少数上访人员的闹事行为，公安机关要及时介入掌握证据，适时依法处理。

　　加大整顿和规范市场经济秩序的力度。重点整治集贸市场、医药市场、食品市场、文化市场、中介市场及金融秩序，建立企业信用征信体系。继续贯彻“安全第一、预防为主”的方针，落实责任制度，加强监督检查，遏制各类生产事故，保障人民群众生命财产安全。

　　三、加强和改进政府机关建设

　　新世纪我们面临着新的情况和任务，对政府工作提出了新的更高的要求。我们要全面贯彻“三个代表”重要思想，坚持与时俱进，切实加强政府自身建设，奋力开创各项工作新局面。

　　一是深入学习贯彻十六大精神，全面落实市委提出的三项重点任务。牢牢把握十六大报告的主题、灵魂和精髓，把学习贯彻“三个代表”重要思想作为中心环节，深刻理解建设中国特色社会主义必须坚持的基本经验，深刻理解全面建设小康社会的奋斗目标和战略部署，深刻理解历史和现实赋予我们的庄严使命。坚持把发展作为执政兴国的第一要务，冲破一切妨碍发展的思想观念，改变一切束缚发展的做法和规定，革除一切影响发展的体制弊端。坚持以经济建设为中心，集中精力抓工业立市、抓农业增效、抓财政翻身。

　　二是营造发展环境，为经济建设服务。深入开展学习长春经济技术开发区海关活动，抓服务促发展。下决心营造出我市良好的典论环境、优惠的政策环境、优质的服务环境、稳定的社会环境和优美的城市环境。认真兑现公开承诺，切实做到主动服务、超前服务、跟踪服务、全程服务和免费服务。进一步落实改审批制为注册制、服务式管理、宽松式经营的特殊政策。以市投资服务中心为基础，筹建政务办公大厅，实行一站式办公，提供一条龙服务。严厉查处干扰经济发展环境的行为，切实保护投资者的合法权益。

　　三是转变工作作风，求真务实、真抓实干。大力推进政务公开，加强政务信息化建设，完善大督查责任制度，改进管理方式，提高行政效率，适应加入WTO后的新形势。真正做到从政为民，厚待百姓，始终把人民群众的根本利益放在首位。主动深入到基层第一线，找准工作切入点，着力解决重点难点问题，创造性地抓好落实。坚决反对和克服官僚主义、形式主义，坚持实事求是，理论联系实际，做到说实话、办实事、重实绩、求实效。

　　四是大力发扬艰苦奋斗作风，勤俭办一切事业。各级政府和广大干部，要清醒地看到激烈的市场竞争给我们带来的严峻挑战，清醒地看到我们肩负任务的艰巨性和复杂性，务必继续保持谦虚、谨慎、不骄、不躁的作风，务必继续保持艰苦奋斗的作风。各级干部特别是领导干部，要不断加强自身修养，磨练艰苦奋斗意志，发扬优良传统，居安思危，率先垂范，在工业立市、建设经济强市的征程中经受新的考验，努力交出优异的答卷。

　　五是坚持依法行政，提高政府决策的法制化、科学化和民主化水平。各级政府要认真接受人民代表大会及其常委会的监督，积极支持人民政协的工作，认真办理人大代表的建议和政协委员的提案，重视社会各界对政府工作的意见和建议。强化执法监督，搞好依法行政考评，规范行政行为。建立杜情民意反馈制度、与群众利益相关的重大事项社会公示制度和社会听证制度。建立和完善决策规则和程序，实行决策论证制和责任制，提高“为民执政，科学理政，依法行政，从严治政”的能力和水平。

　　六是深入开展反腐败斗争，建设廉洁高效政府。严格执行中央和省、市有关领导干部廉洁自律的制度规定，认真落实党风廉政建设责任制，规范领导干部的从政行为。各级领导干部要自重、自省、自警、自励，用好手中的权，尽好自己的责，办好人民的事，始终做到清正廉洁，自觉地与各种腐败现象作斗争。下力量纠正部门和行业不正之风．解决好群众反映强烈的突出问题。坚持标本兼治、综合治理的方针，逐步加大治本的力度。加强教育，发展民主，健全法制，强化监督，创新体制，把反腐败寓于各项重要政策措施之中，从源头上预防和解决腐败问题。形成行为规范、运转协调、公正透明、廉洁高效的行政管理体制。

　　各位代表，明年是本届政府的最后一年，做好各项工作，对实现本届政府的预期目标至关重要。我们要按照“三个代表”的要求，在市委的领导下，坚定信心，开拓进取，狠抓落实，迎接挑战，为实现建设经济强市目标和社会全面进步而努力奋斗！
